# Truth-Telling to Palliative Care Patients from the Relatives’ Point of View: A Türkiye Sample

**DOI:** 10.3390/healthcare13141644

**Published:** 2025-07-08

**Authors:** İrem Kıraç Utku, Emre Şengür

**Affiliations:** 1 Department of Internal Medicine, Division of Internal Medicine, Tekirdağ İsmail Fehmi Cumalıoğlu City Hospital, 59030 Tekirdağ, Türkiye; 2 Department of Internal Medicine, Division of Geriatrics, Tekirdağ İsmail Fehmi Cumalıoğlu City Hospital, 59030 Tekirdağ, Türkiye; 3 Palliative Care Unit, Sancaktepe Prof. Dr. İlhan Varank Training and Research Hospital, 34785 Istanbul, Türkiye

**Keywords:** palliative care, caregiver, resilient responses, spirituality

## Abstract

**Aim**: This study aimed to explore the attitudes of family caregivers toward truth-telling practices in palliative care in Türkiye, a Muslim-majority context where disclosure is often mediated by relatives. **Methods**: Using a convergent parallel mixed-methods design, data were collected from 100 unpaid family caregivers of terminally ill patients at a palliative care unit. Quantitative data were gathered via a structured questionnaire, and qualitative data through in-depth interviews with a purposively selected subsample of 10 participants. Chi-square tests were used to analyze associations, and *p* < 0.05 was considered statistically significant. **Results**: The mean age of caregivers was 47.4 ± 16.5 years, 67% were female. Notably, 67% of participants did not prefer that the patient be informed of irreversible deterioration, while 71% stated they would want to be informed if they were in the patient’s position (*p* < 0.05). Most preferred a multidisciplinary disclosure process involving physicians, psychologists, and spiritual counselors. Qualitative analysis revealed four themes: emotional conflict, protective family-centered decision-making, spiritual readiness for death, and preference for multidisciplinary communication approach. The participants expressed cultural concerns about psychological harm to the patient and emphasized the family’s role as emotional guardians. **Conclusions**: The findings highlight a gap between caregivers’ attitudes when acting as family members versus imagining themselves as patients. These results underscore the critical need for culturally sensitive and family-inclusive communication strategies in palliative care settings.

## 1. Introduction

Due to scientific and technological advancements both globally and in Türkiye, as well as technological advances, life expectancy has increased. This rise in life expectancy has, in turn, led to a growing prevalence of chronic diseases [1,2]. Today, Healthcare efforts are not solely focused on curing diseases, but also on enhancing quality of life. Rising life expectancy and the increasing incidence of chronic diseases have increased the need for palliative care.

The World Health Organization defines palliative care as “an approach based on improving the quality of life of patients and their relatives facing problems resulting from a life-threatening disease by preventing and eliminating all physical, psychosocial, and spiritual problems, especially pain, through early detection and effective assessments [3].” At this point, palliative care is a system of care that aims to improve quality of life in the face of progressive and terminal disease. Palliative care views living and dying processes as normal parts of life. In palliative care, death is neither delayed nor hastened. The primary goal is not to extend life span, but to improve quality of life. Emphasis is placed on supporting patients and their relatives during the grieving process [4]. The current structure necessitates a multidisciplinary and interdisciplinary approach.

The palliative care service has a comprehensive team of physicians, dietitians, nurses, psychologists, social workers, and spiritual counselors. This system also encompasses three interacting groups: patients, their family caregivers, and healthcare professionals—each coexisting and intertwined in a complex relational network. In the process of breaking bad news, legal and ethical considerations also come to the fore. Telling the truth, a basic rule of Western medicine, is a globally shared moral stance [5].

It is essential to ensure that the patient’s individual rights are respected and that he or she has a say in medical decisions. The patient’s autonomy is linked to his or her informedness and voluntariness. According to the principle of respect for patient autonomy, the physician is obliged to respect the patient’s right to make decisions and act in accordance with his or her values [6]. When it comes to bad news in palliative care, it is essential to inform patients and their relatives about the progression of the disease, potential complications, and the possibility of death. Breaking bad news is a communication process among the patient, their relatives, and healthcare professionals. Honest disclosure of the patient’s diagnosis, prognosis, and terminal stage is considered essential to end-of-life preparation. Yet, concealment of the truth is common in many cultures. In contrast to Western culture, the disease is often regarded as a collective family matter, especially in Asian and Muslim cultures. In many cultures, families and physicians may choose to withhold the truth about their patient’s disease. Usually, the truth is revealed to an authorized family member who has become the guardian, and then the physician and family member argue about whether to reveal the truth to the patient. Most often, the truth is not explained for fear that the patient will become distressed and be in despair [7]. In Türkiye, where cultural norms reflect a synthesis of Muslim identity and Asian collectivist values, there is a tendency to withhold medical information from the patient. Instead, the disclosure process is often mediated through family members, who act as intermediaries in communicating with healthcare professionals [7].

In clinical practice, questions such as how, by whom, to whom, and in what sequence bad news should be delivered often lead to uncertainty and emotional strain for both healthcare professionals and patients’ family members. This study aims to explore the attitudes of family caregivers of patients receiving palliative care in Türkiye regarding being informed of potential disease progression, clinical deterioration, and the process of dying. Additionally, the study examines their perspectives on being notified about significant changes in diagnosis or disease status. The primary aim of this study was to identify prevailing attitudes within the cultural and religious context of Turkish society shaped by Islamic values. By investigating the perceptions of informal caregivers of terminally ill patients, this study seeks to provide insights that may inform and support healthcare professionals in the development of culturally sensitive communication strategies for delivering bad news.

## 2. Materials and Methods

This research was conducted using a convergent parallel mixed-methods design, which integrates both quantitative and qualitative data collection and analysis techniques.

### 2.1. Type, Place, and Time of the Study

The cross-sectional study was conducted at the annex building of Health Sciences University Sancaktepe Martyr Prof. Dr. Ilhan Varank Training and Research Hospital between 18 November 2021 and 10 April 2022 (six months).

### 2.2. Study Population and Sampling

The study sample consisted of family members aged 18 and older, who were responsible for the care of patients hospitalized in the palliative care service at the annex building of the mentioned hospital between the specified dates, and who voluntarily agreed to participate in the study without receiving payment for care. Among the 143 family caregivers approached during the study period, 100 agreed to participate, resulting in a response rate of 69.9%. Additionally, all patients cared for by the family members who constituted the study sample had life-threatening illnesses (malignancies, cerebrovascular diseases, cardiovascular diseases, chronic obstructive pulmonary diseases, other progressive neurological diseases). All participants identified as Muslim.

### 2.3. Data Collection Tools

The research data were collected using the “Personal Information Form” and the “Attitude Questionnaire Toward Bad News Breaking Processes” through face-to-face interviews lasting approximately 10–12 min in a quiet and private setting.

*Personal Information Form* This form was developed by researchers based on the results of a literature review and questions individuals about information such as their age, gender, level of education, the duration of caregiving for the patient’s relative, and the presence of any chronic diseases [8]. Attitude *Questionnaire Toward Bad News Breaking Processes:* The form was created by researchers—two experts with experience in palliative care clinics, a psychologist and a spiritual counselor—as a result of a literature review [9,10,11]. Based on this review, the questionnaire items were constructed to reflect relevant themes. The draft version was evaluated for content validity by an expert panel consisting of four professionals, and necessary linguistic revisions were made. A pilot test was conducted with ten caregivers to assess the clarity and feasibility of the questionnaire. Following this, it was administered face-to-face to participants after obtaining written informed consent. The average completion time was approximately 10–12 min. The final version of the questionnaire consisted of ten items organized into two subgroups. Four questions were in yes/no format, and the remaining six were multiple choice. The first four questions were answered from the perspective of a caregiver for a terminally ill patient. The remaining questions explored participants’ anticipated attitudes if they were to personally experience a serious illness (Table 1). As the questionnaire was designed solely for the purpose of evaluating our own study population and not intended as a psychometric tool for broader use, validity and reliability analyses were not performed.

### 2.4. Evaluation of the Research

In the G* Power analysis, when the effect size was accepted as 0.5 and alpha was 0.05, it was deemed necessary to include a minimum of 54 people to obtain 95% power. The study included 100 participants who were caring for their family member patients with terminal illness in a palliative care unit and volunteered after obtaining both their verbal and written consent.

### 2.5. Qualitative Phase

During the qualitative phase, a subsample of 10 participants was selected from the original quantitative sample using purposive sampling. The participants were chosen to ensure variation in education level, age, and caregiving duration, aiming to maximize both the representativeness and depth of the qualitative findings. In-depth interviews were conducted with those who volunteered to participate in the qualitative phase.

The data were gathered through semi-structured interviews using a form created by the researchers. The interview guide was critiqued by two specialists in qualitative health research and palliative care. The actual interviews took place in a quiet, private room at the hospital. The duration of each interview was about 20–30 min, and each was recorded with permission from the participants. Qualitative interviews typically take longer than quantitative ones, as the participants are given ample time to respond in depth.

The qualitative data were transcribed verbatim, and thematic content analysis was applied. The transcripts were independently coded by two researchers, and any differences resolved in a consensus meeting. Analysis was aided using MAXQDA 24 software.

To ensure the trustworthiness of the qualitative findings, several strategies were implemented: Credibility was established through member checking and peer debriefing. Transferability was supported by providing a detailed description of participants and the study context. Dependability and confirmability were ensured through maintaining an audit trail and applying analyst triangulation to preserve consistency in coding.

In line with the convergent mixed-methods design, quantitative and qualitative results were analyzed separately and then integrated during the interpretation phase to comprehensively capture family caregivers’ perspectives on truth-telling in palliative care.

### 2.6. Ethical Aspects of the Research

The present study was started after approval was obtained by the Ethics Committee (17 November 2021; Number 2021-230) of Health Sciences University Sancaktepe Martyr Prof. Dr. Ilhan Varank Training and Research Hospital. Prior to participation, both verbal and written informed consent was obtained from all participants. The researcher informed the participants about the aim and design of the study and advised them that they could withdraw from the study at any time. The study was conducted in compliance with the principles of the Declaration of Helsinki.

### 2.7. Statistical Analysis

For the statistical analysis, the frequency (*n*) and percentage (%) values and the mean (Mean), standard deviation (SD), minimum (Min) and maximum (Max) values were calculated. Shapiro–Wilk test was performed to evaluate whether the data were normally distributed, and nonparametric statistical techniques were used since the data were not normally distributed. Information obtained through the questionnaire was entered into the statistical program IBM Statistical Package for the Social Sciences 25.0 (IBM SPSS 25.0), and the differences between the groups were evaluated using the chi-square test. A value of *p* < 0.05 was considered significant.

## 3. Results

A total of 100 volunteer caregivers participated in the study. Of these, 67 were female and 33 were male. The majority were primary school graduates. The mean age was 47.4 ± 16.5 years, with the most common age group being 50–60 years. Fifty percent of caregivers had been providing care for up to three months, while 35% had been providing care for more than 12 months. Additionally, 28% of caregivers reported having a chronic illness themselves (Table 2).

According to the answers to the questions, the questionnaire was designed to answer considering the caregiver role of the patient’s relative. 67% of the participants did not want their patients to be informed about the possible irreversible deterioration and the possible course of dying, but 96% wanted them to be informed themselves as patient companions. 47% wanted to be informed by the physician, psychologist, and spiritual counselor as a team, and 63% wanted only the patient companion to be informed (Table 3).

The second part of the questionnaire includes questions that asked participants to reflect on how they would respond if they personally experienced a serious illness. A total of 71% indicated that they would want to be informed about their own condition, and 82% stated that they would want their caregiver or family member to be informed as well. Furthermore, 46% preferred that such information be delivered by a multidisciplinary team consisting of a physician, psychologist, and spiritual counselor. Notably, 32% of participants preferred that the caregiver be informed first, followed by themselves as the patient (Table 4).

When participants were asked how they would react if they experienced a possible disease course, 94% said they would accept the situation (disease) and 6% said that they would not. Of the group that said they would accept the disease, 7.4% said they would not accept treatment, and 92.6% said they would seek treatment with the healthcare team. Of the group that stated they would not accept the disease, 16% stated they would not accept treatment. 84% said they would seek treatment from other hospitals. When participants were asked what they would most need support for if they entered a possible disease course, 65% said they would need medical care, spiritual counseling, and psychological support.

While most participants did not want their patients to be informed, they indicated that they would want to be informed if they assumed they were patients. There was a significant difference between questions 1 and 5 (*p* < 0.05). Participants’ responses toward the role of the patient and the patient’s relatives were similar in their opinion that the patient’s relatives should be informed. There was no significant difference between questions 2 and 6 (*p* > 0.05) (Table 5). Although female participants made up two-thirds of the sample, chi-square analyses revealed no statistically significant gender differences in responses to key questions (*p* > 0.05).

There was no significant difference between the gender of the participants and the presence of chronic disease and their responses to questions 1, 2, 5, and 6 (*p* > 0.05) (Table 6).

### 3.1. Qualitative Results

Analysis of the qualitative data uncovered four key themes that describe the sociocultural emotional aspects of truth telling in palliative care. Participants’ worrying thoughts that patients may undergo emotional collapse tended to be a predominant issue for them, which participants identified as Emotional Conflict and Anxiety. This conflict often caused caregivers to restrict information in order to safeguard their loved ones from trauma. The second theme, Protective Instinct and Family Centredness, reveals the cultural expectation of family members as emotional protectors and primary decision-makers who tend to bypass the sovereignty of the patient for the sake of family balance. Participants’ understanding of dying was reflective in the final two themes which were more outward focused and deeming the knowledge of dying to be an important requirement for spiritual preparations and a meaningful closure was termed as Perception of Death and Spiritual Readiness. Lastly, the most pronounced aspect in the discourse was the lack of physicians and psychologists as well as spiritual counselors willing to provide emotional and spiritual support to address the bad news, which participants termed as Preference for Multidisciplinary Communication. These themes illustrate how caregivers, within their sociocultural context in Türkiye, navigate the challenges of truth telling in palliative care (Table 7).

### 3.2. Theme 1: Emotional Conflict Around Telling the Truth

“Part of me does not want to tell the truth since it may lead to my patient collapsing. They are already in a very fragile state, and this would be the final blow.” (P3)

“Knowing the patient deserves to know what is going on is one thing, but I’m petrified as well. What if hearing the truth causes them to give up completely?” (P7)

“It is wrong to keep the secret, but it feels impossible to actually say it.” (P2)

Participants showed a significant level of emotional conflict with telling the truth. By telling the truth, they had to provide disclosure which, in this case, they did not want to give. Their mind was dominated by the possibility of a breakdown dense with fear. There seems to be an emotional load caregivers bear—where silence, in this context, is not apathy or indifference but rather a desire to protect on account of anxiety and guilt.

### 3.3. Thematic Category 2: Protective Family Instincts Along with Family-Centered Decision-Making

“It is the doctor who will tell us, and we will decide how and when to inform the patient.” (P1)

“Family does everything and knows everything and acts with the patient, for the patient. That is how it is done in our culture.” (P4)

“I hold emotional protection over them. What if they can’t handle it?” (P6)

Everyone expressed that the family functions as a buffer to the patient and the healthcare provider, revealing the triadic system dynamic. The caregivers perceived their role as companions to their patients and at the same time gatekeepers to the information which the patient ought to be availed. This gives a glimpse of a cultural framework where strong family unit is viewed as a source of protection towards an individual’s vulnerability especially from the Eastern and Muslim world.

### 3.4. Theme 3: Understanding Death in Context of Dignity

“People should be informed so that they can take their leave appropriately. Everything needs to be communicated.” (P10)

“Every individual seeks to prepare for death; they say their prayers and leave a will.” (P5)

“When they aren’t aware, I feel like their life is unfinished.” (P2)

This theme demonstrates a wish for peace, particularly in the context of emotional and spiritual closure. Many participants noted that telling the truth helps patients prepare for death, fulfill their wishes, and maintain dignity. The assumption highlighted, in this case, was the existence and acceptance of one’s condition enables the person to meaningfully and purposefully complete life.

### 3.5. Thematic Focus 4: Anticipation of Multidisciplinary Collaboration

“I believe treatment requires more than a doctor. There is always need for a psychologist and even clergy. Each role is critical.” (P6).

“It’s not as scary if a doctor, psychologist, or even an imam explains things together,” (P4).

“Patients cope better, even with bad news, when it is accompanied by ‘support’ and ‘good intention’.” (P9).

It can be interpreted that participants strongly preferred a multidisciplinary approach when addressing the bad news. The inclusion of psychological and spiritual intervention was emphasized repeatedly. This suggests that families in Türkiye possess fundamental holistic expectations regarding end-of-life care as they consider supportive elements to be crucial for guiding patients through emotional processing.

## 4. Discussion

Receiving a diagnosis marks the beginning of a new identity for the individual as a patient. Various role changes, new responsibilities, and physical-emotional consequences of the disease occur for the patient with the diagnosis. The disease process is a time when many changes occur not only for the patient but also for the patient’s relatives [12]. In this context, palliative care is a field whose importance is increasingly recognized due to the growing population, longer life expectancy, and increase in chronic diseases. Palliative care services are inpatient services with a high likelihood of treatment complications and end-of-life outcomes [13]. Confronting dying can be traumatic for the patient and the patient’s relatives. At this point, it becomes important in palliative care for the patient, patient’s relatives, and staff to be able to support each other as a team in breaking bad news [14].

The literature suggests that the reluctance of healthcare professionals to tell the patient the whole truth and the associated loss of motivation impair the informing process [15,16]. Nowadays, the reluctance of physicians to inform the patient is changing [17]. However, withholding information from the patient is still common in our culture, especially at the request of the patient’s relatives. In response to the question posed in our study—“Would you want your patient to be informed about the possibility of irreversible deterioration and dying?”—67% of participants answered “No.” Based on these responses, it was clear that most of the patients’ relatives did not want the patients to be informed during the process of receiving bad news. This situation reflected the relatives’ fear of the difficulties that the patients will have in confronting the current news, accepting it, and coping with it. The literature presents varying results depending on cultural context. In one comparative study, nearly all African American and European American relatives believed that patients should be fully informed of their diagnosis (87% and 89%, respectively), whereas only 47% of Korean Americans and 65% of Mexican Americans supported full disclosure [18]. A study conducted in Iran similarly found that 75.5% of patients’ relatives did not want the terminal nature of the disease to be disclosed to the patient [19].

When people take on different roles, their reactions to events also change. In our study, the participants were also asked to consider the scenario from the patient’s perspective. In response to the question, “If you were the patient, would you want to be informed about your own possible irreversible deterioration and dying?”, 71% of participants answered “Yes.” The majority of participants wanted to be informed about the adverse course of treatment. A study conducted in Türkiye examining the preferences and expectations of healthy individuals in the event of a terminal illness diagnosis found that most participants wished to be fully informed about their condition and the diagnosis [20]. To our knowledge, no previous study in the literature has examined how caregivers respond to disclosure-related questions when asked to adopt both the roles of caregiver and patient. In our study, the participants demonstrated significantly different attitudes toward disclosing information to the patient depending on whether they imagined themselves as the caregiver or as the patient. While participants expressed a desire to be informed about their own illness if they were the patient, they did not support informing the patient when they were acting in the role of a caregiver. The present result shows that participants’ role in the process of receiving bad news influences their attitudes toward the informing process.

Guardianship, while originally a legal concept, often takes on a broader cultural meaning in Eastern and Muslim societies, where the well-being of the family is frequently prioritized over individual autonomy [19]. In response to the question, “As the patient’s relative or caregiver, would you prefer to be informed before the patient is told about their condition?”, 96% of participants answered “Yes.” This answer shows that almost all participants would like to be informed about the course as a patient companion. Chronic illness introduces new dynamics into individuals’ lives, requiring them to adapt to evolving responsibilities and roles. For those who serve as caregivers, this often means balancing their personal lives with the emotional and physical demands of caregiving. The onset of serious illness often introduces the roles of ‘patient’ and ‘patient companion’ into individuals’ lives, fundamentally altering family dynamics and decision-making responsibilities. Individuals are often required to assume new roles and responsibilities, particularly when acting as caregivers for terminally ill relatives, which includes navigating complex emotional, ethical, and decision-making duties. In this way, patient relatives can become active decision-makers for their patients. This phenomenon can be described as emotional guardianship—a culturally rooted role emotionally adopted by patient companions after the onset of serious illness [20]. The present result also points to this situation.

Eighty-three percent of participants answered “Yes” to the question, ‘If you were in your patient’s place, would you want your patient companion to be informed about your possible irreversible deterioration and dying?’ This suggests that the majority of respondents preferred their relatives to be informed about the potential progression of their illness. Individuals diagnosed with life-threatening illnesses often express a desire to spend their remaining time in the presence of loved ones, particularly family members, in order to achieve emotional closure and bid farewell [21]. These results indicate that participants consistently preferred informing family members, regardless of whether they viewed the situation from a caregiver or patient perspective. In one study, patients reported that the involvement of their family members played a crucial role in helping them make informed treatment decisions. Patients also noted that their loved ones influenced both the decision-making process and the decisions themselves; however, the final choice was ultimately left to the patients [22]. Also, in another study exploring health professionals’ views and experiences, it was suggested that the presence of family members is crucial when patients receive bad news [23]. It is considered acceptable for patients to seek support from family members when making decisions regarding their own illness.

No statistically significant differences were found in responses to the questions “Would you want your patient to be informed about the possibility of irreversible deterioration and dying?” and “As the patient’s relative or caregiver, would you prefer to be informed about this before the patient is told?” based on participants’ gender or the presence of a chronic illness. This was consistent across both the patient and companion role perspectives. Consistent with previous research, gender was not found to be associated with participants’ attitudes toward receiving information about serious health conditions [24]. The predominance of female caregivers in the study reflects broader sociocultural norms in Türkiye, where caregiving responsibilities are commonly assumed by women. This cultural norm may have influenced both participation patterns and the caregiving attitudes observed in our study.

To our knowledge, there are no existing studies in the literature examining how caregivers with chronic illnesses behave in relation to informing their patients. However, having personally experienced illness may increase individuals’ awareness of the importance of being informed about their own health status in the future. According to a study, the participants demonstrated increased awareness of the need for empathic and transparent communication from healthcare professionals during their own illness experiences. Individuals who have experienced their own illness may be more willing to accept inadequate information being provided to others in the future. We hypothesized that attitudes toward information seeking may change if a person has a chronic disease, but no difference was found.

Because the person who provides the information and the way they provide it greatly influence the process that follows, various studies are being conducted on this topic and attempts are being made to develop different information techniques [25]

Each person has a different way of dealing with the problem, which affects their decisions when it comes to communicating bad news. Telling the truth is not just about informing about impending death. It is also about how physicians and other healthcare professionals can support patients’ hope by tailoring the delivery of the “truth” to the individual’s preferences [26,27].

In our study, the following question was asked: “Who do you think should inform the family about such serious health news?” When participants responded from both the perspective of the patient’s relatives and that of the patient, they similarly preferred that the physician, psychologist, and spiritual counselor provide the information together. This indicates that participants desire comprehensive coverage in terms of informational content, as well as psychological and spiritual support when receiving bad news. At this point, it becomes evident that breaking bad news involves not only the delivery of information but also the need to assess psychological and spiritual well-being before and after the disclosure. The SPIKES model offers a framework for breaking bad news and highlights the importance of monitoring psychosocial and emotional responses. This model recommends a sensitive approach to managing patients’ emotional reactions following the disclosure [25]. In the NCCN guideline, the aim is to provide psychosocial support after the delivery of bad news; to help patients and their families cope with the process. It also emphasizes the importance of offering moral support [28].

As an expression of the cultural tendency of the family to protect the person in our country, 63% of the participants answered the question “In what order would you want to be informed about the possible irreversible deterioration and dying? “with “Only the patient companion should be informed.” In the case where both the patient and the patient companion were informed, 27% of participants wanted the patient companion to be informed first. Only 5% preferred that the patient be informed before the companion. This reflects the role patient companions often assume in informing the patient after first receiving the information themselves. The patient companions want to have a decisive say in whether and how they are informed by learning the process first. Studies have shown that patient companions tend to withhold some information from the patient, especially in Eastern cultures [24]. In this situation, the patient companion assumes great responsibility.

The patient companion serves both as support during the delivery of bad news and as an “emotional guardian.” When participants were asked the same question while imagining themselves as the patient, 32% stated that the companion should be informed first, followed by the patient. On the other hand, 26% of participants said “First the patient should be informed, then the patient companion.” As the participants shifted roles, the proportion of those who preferred not to be informed in case of illness decreased.

Participants were asked to imagine themselves as patients and indicate how they might react to receiving such news. In total, 87% of participants responded “I accept the information given; I plan the course of treatment and care with the healthcare team.” This result indicates that the majority of participants have an accepting attitude toward the process of delivering bad news. In a recent study, when caregivers imagined themselves as cancer patients, they showed greater willingness to participate in non-standardized drug trials, whereas they were more hesitant about involving their own patients in such trials. It was observed that participants reached acceptance about their own illness more quickly [29]. Considering that the current study was conducted with the patient companions of the palliative care service, it was hypothesized that the disease and acceptance processes of the participants influenced the current response. In total, 65% of participants stated that they would like to receive support in the fields of “Medical Care, Spiritual Counseling, and Psychological Support.” The present result suggests that the majority of participants need psychological support and spiritual counseling in addition to medical care during the course of disease and possible adverse treatments. This finding shows that psychologists and spiritual counselors may be important sources of support for patients and their relatives in palliative care [30].

Participants’ issues regarding disclosure primarily focused on possible emotional damage. The issue of emotional conflict in the caregivers regarding truth-telling is deeply concerning. Most of them thought that telling patients about the terminal nature of their illness would make them go into either despair or a deep psychological collapse. Such reluctance is supported by earlier studies which highlight the emotional and ethical strain families endure while trying to tell the truth [5,14,19]. The need to address these problems culturally is even greater due to the focus on family interdependence and emotional protection, which tend to trump individualism [12].

To a large extent, caregivers positioned themselves as emotional guardians between the physician and the patient, which allowed them to control what information was shared. This combination reinforces the notion of “emotional guardianship,” which views the family, rather than the individual patient, as the key decision-maker, and is most evident in Muslim and collectivist cultures [6,12,18]. These results also align with Turkish and Iranian findings, indicating that family decision-making is dominant and that the truth is not always disclosed [10,19,29].

Participants emphasized the deeply spiritual and emotional impacts of the truth, especially in relation to death. Many caregivers believed that terminally ill patients needed to be informed in order to complete their life journey spiritually and emotionally. This aligns with the research which suggests that knowing about dying has the potential to provide closure, last wishes, and peace of mind for patients and families [21,27,30]. The notion of death with dignity is a common framework in the literature and highlights the ethical case for honest, open communication that has also been documented [5,23].

Another key finding was the overwhelming need for multidisciplinary communication approaches in palliative care. Participants expressed the need for an integrated approach where physicians worked together with psychologists and spiritual caregivers. This is in line with other palliative care approaches that support the need for biopsychosocial–spiritual models of care [3,28]. More formal approaches like the SPIKES model also highlight the need for interprofessional communication and relations within the healthcare team when giving serious information [25,26]. These participants fit international recommendations which emphasize the need for trained professionals from different disciplines to provide care that is sensitive to the emotions and culture of patients.

Overall, these qualitative findings demonstrate that telling the truth in palliative care is not simply a matter of conversation but, rather, a multi-layered emotion steeped in culture. In the Turkish context, families bear two responsibilities simultaneously: safeguarding the essence of their loved one while struggling with personal doubts and assumptions. These findings further highlight the importance of culturally appropriate communication strategies and multidisciplinary approaches to adequately support both families and healthcare professionals.

## 5. Limitations

The strength of this study lies in its use of a mixed-methods approach, integrating both quantitative and qualitative data collection and analysis techniques. Another notable strength is that participants were encouraged to reflect on the roles of both the patient and the caregiver during their responses. While a substantial body of literature exists on attitudes toward palliative care, this study’s distinctive contribution lies in its focus on an Eastern, Muslim-majority Turkish population, contrasting with the predominantly Western contexts explored in previous research. To enhance the comprehensiveness of the findings, future research could include a larger dataset and a broader participant pool, incorporating diverse groups such as patients, individuals not directly involved in palliative care, and other family members. Another potential limitation of this study is the gender imbalance among participants, as female participants significantly outnumbered their male counterparts. This disparity may limit the generalizability of the findings.

The questionnaire used in this study consisted of original, non-validated items specifically designed for this research. While this limits the generalizability of the results, it still offered meaningful and context-specific insights into caregivers’ experiences. The results may serve as a preliminary step toward the development of standardized instruments in future studies.

## 6. Conclusions

This mixed-method study illustrates the multi-layered, culturally embedded nature of truth-telling in palliative care from the perspective of patients’ relatives in Türkiye. The findings reveal the psychological paradox and protective instincts rooted in cultural responsibility, family duty, and emotional guardianship.

The results validate that relatives take a pivotal role in controlling information as access intermediaries between the patient and the healthcare staff. This constellation of family power dynamics in a culturally collectivist and Muslim society challenges Western notions of autonomy in individualistic medical ethics and raises ethical questions about medical authority. Yet, a significant proportion of participants emphasized the need to maintain dignity along with spiritual preparation for death and reaffirmed the importance of truth telling. The participants’ wish for bad news to be given by a multi-disciplinary team speaks to their desire for integrated and compassionate care. Due to the emotional, spiritual, and ethical burden of delivering bad news in the palliative care context, culturally appropriate communication frameworks and supportive structures for caregivers are essential. Courses for healthcare education and training should consider family dynamics while incorporating interprofessional collaboration that involves psychologists and spiritual care providers. Perhaps the most compassionate and practical approach to communicating truth during end-of-life care will be guided by a biopsychosocial-spiritual paradigm.

## Figures and Tables

**Table 1:** Attitude questionnaire toward bad news breaking processes.

**1.**	Would you want your patient to be informed about the possibility of irreversible deterioration and dying?
**2.**	As the patient’s relative/caregiver, would you prefer to be informed about this before the patient?
**3.**	Who do you think should inform the patient about such serious health news?
**4.**	In what order should the news be shared?
**5.**	If you were in your patient’s place, and the current course of treatment was the same for you, would you want to be informed about your own possible irreversible deterioration and dying?
**6.**	If you were in your patient’s place, would you want your patient companion to be informed about your possible irreversible deterioration and dying?
**7.**	From whom would you want to be informed about your possible irreversible deterioration and dying?
**8.**	In what order would you want to be informed about the possible irreversible deterioration and dying?
**9.**	How do you think you would emotionally react to receiving such news?
**10.**	In what areas would you want support after receiving such news?

**Table 2:** Demographic characteristics of the participants (*n* = 100, Türkiye, 2025).

Variables	Levels of Variables	*n*	%
Gender	Male	33	33
	Female	67	67
Education	Primary education	59	59
	Secondary education	22	22
	University	18	18
	Master’s/PhD	1	1
Duration of care	0–3 months	50	50
	3–6 months	9	9
	6–12 months	6	6
	>12 months	35	35
Presence of a chronic disease	Yes	28	28
	No	72	72

**Table 3:** Attitude assessment survey questions toward bad news breaking process (*n* = 100, Türkiye, 2025).

Questions	Responses	*n*	%
Would you want your patient to be informed about the possibility of irreversible deterioration and dying?	Yes	33	33
	No	67	67
As the patient’s relative/caregiver, would you prefer to be informed about this before the patient?	Yes	96	96
	No	4	4
Who do you think should inform the family about such serious health news?	Physician	30	30
	Psychologist	2	2
	Physician/Psychologist	21	21
	Physician/Psychologist/Spiritual Counselor	47	47
In what order should the news be shared?	Inform only the patient	1	1
	Inform only the patient companion	63	63
	Inform the patient first, then the patient companion	5	5
	Inform the patient companion first, then the patient	27	27
	Do not give out any information	4	4

**Table 4:** Attitude Assessment Survey Questions Toward Bad News Breaking Process (*n* = 100, Türkiye, 2025).

Questions	Responses	*n*	%
If you were in your patient’s place, and the current course of treatment was the same for you, would you want to be informed about your own possible irreversible deterioration and dying?	Yes	71	71
	No	29	29
If you were in your patient’s place, would you want your patient companion to be informed about your possible irreversible deterioration and dying	Yes	83	83
	No	17	17
From whom would you want to be informed about your possible irreversible deterioration and dying?	Physician	29	29
	Psychologist	3	3
	Spiritual Counselor	1	1
	Physician and Psychologist together	21	21
	Physician, Psychologist and Spiritual Counselor together	46	46
In what order would you want to be informed about the possible irreversible deterioration and dying?	Inform only the patient	12	12
	Inform only the patient companion	23	23
	Inform the patient first, then the patient companion	26	26
	Inform the patient companion first, then the patient	32	32
	The patient and the patient companion should not be informed	7	7
How do you think you would emotionally react to receiving such news?	I accept the information given to me; I plan the course of treatment and care with the healthcare team.	87	87
	I accept the information given to me; I decline further treatment and care	7	7
	I do not accept the information given to me; I refer to other hospitals and specialists	5	5
	I do not accept the information given to me; I decline further treatment and care, including at other hospitals.	1	1
In what areas would you want support after receiving such news?	Medical Care	9	9
	Psychological support	3	3
	Medical Care and Spiritual Counseling	2	2
	Medical Care and Psychological Support	20	20
	Spiritual Counseling and Psychological Support	1	1
	Medical Care, Spiritual Counseling and Psychological Support	65	65

**Table 5:** Relationship between Question 1 and Question 5 and Question 2 and Question 6 (*n* = 100, Türkiye, 2025).

Question 5			Question 5	*p* Value
Yes				No
Question 1	Yes	28	5	0.032 *
	No	43	24	
**Question 6**			**Question 6**	***p*** **Value**
**Yes**				**No**
Question 2	Yes	80	16	0.089
	No	2	2	

* Chi-square test, *p* < 0.05 was considered significant. Question 1: Would you want your patient to be informed about the possibility of irreversible deterioration and dying? Question 2: As the patient’s relative/caregiver, would you prefer to be informed about this before the patient? Question 5: If you were in your patient’s place, and the current course of treatment was the same for you, would you want to be informed about your own possible irreversible deterioration and dying? Question 6: If you were in your patient’s place, would you want your patient companion to be informed about your possible irreversible deterioration and dying?

**Table 6:** Distribution of responses given to questions by gender and chronic disease status (*n* = 100, Türkiye, 2025).

Gender					Chronic Disease				
		Female %	Male %	*p* Value			Present %	Absent %	*p* Value
Question 1	Yes	22	11	0.96	Question 1	Yes	8	25	0.55
	No	45	22			No	20	47	
Question 2	Yes	64	32	0.72	Question 2	Yes	26	70	0.31
	No	3	1			No	2	2	
Question 5	Yes	49	22	0.50	Question 5	Yes	19	52	0.66
	No	18	11			No	9	20	
Question 6	Yes	53	29	0.28	Question 6	Yes	22	60	0.57
	No	14	4			No	6	12	

Chi-square test, *p* < 0.05 was considered significant. Question 1: Would you want your patient to be informed about the possibility of irreversible deterioration and dying? Question 2: As the patient’s relative/caregiver, would you prefer to be informed about this before the patient? Question 5: If you were in your patient’s place, and the current course of treatment was the same for you, would you want to be informed about your own possible irreversible deterioration and dying? Question 6: If you were in your patient’s place, would you want your patient companion to be informed about your possible irreversible deterioration and dying?

**Table 7:** Themes and subcodes from qualitative analysis.

Theme	Subcodes	Participants
1. Emotional Conflict and Anxiety	Fear of emotional collapse, hesitation to confront the truth	P3, P2, P7
2. Protective Instinct and Family-Centeredness	Emotional guardianship, family as the decision-maker	P1, P4, P6
3. Perception of Death and Spiritual Readiness	Desire for farewell, spiritual preparation, dignity in dying	P5, P9, P10
4. Preference for Multidisciplinary Communication	Psychological support, spiritual counseling, team-based approach	P6, P7, P8

## Data Availability

All data were obtained from the Web of Science database.
